# Impact of stated barriers on proposed warfarin prescription for atrial fibrillation: a survey of Canadian physicians

**DOI:** 10.1186/1477-9560-12-13

**Published:** 2014-06-23

**Authors:** Stuart G Nicholls, Jamie C Brehaut, Rubab G Arim, Kelly Carroll, Richard Perez, Kaveh G Shojania, Jeremy M Grimshaw, Roy M Poses

**Affiliations:** 1 Department of Epidemiology and Community Medicine, University of Ottawa, Ottawa, Ontario, Canada; 2 Ottawa Hospital Research Institute, General Campus, Clinical Epidemiology Program, Centre for Practice-Changing Research (CPCR), 501 Smyth Road, Ottawa, Ontario, Canada; 3 ICES uOttawa, Ottawa Hospital Research Institute, Ottawa, Ontario, Canada; 4 Sunnybrook Research Institute, Sunnybrook Health Sciences Centre, Toronto, Canada; 5 Department of Medicine, University of Ottawa, The Ottawa Hospital, General Campus, 501 Smyth Road, Ottawa, Ontario, Canada; 6 Foundation for Integrity and Responsibility in Medicine, Warren, Rhode Island, USA; 7 Department of Medicine, Warren Alpert Medical School of Brown University, Providence, Rhode Island, USA

## Abstract

**Background:**

Atrial fibrillation (AF) is a common cardiac arrhythmia, and leading cause of ischemic stroke. Despite proven effectiveness, warfarin remains an under-used treatment in atrial fibrillation patients. We sought to study, across three physician specialties, a range of factors that have been argued to have a disproportionate effect on treatment decisions.

**Methods:**

Cross-sectional survey of Canadian Family Doctors (FD: n = 500), Geriatricians (G: n = 149), and Internal Medicine specialists (IMS: n = 500). Of these, 1032 physicians were contactable, and 335 completed and usable responses were received. Survey questions and clinical vignettes asked about the frequency with which they see patients with atrial fibrillation, treatment practices, and barriers to the prescription of anticoagulants.

**Results:**

Stated prescribing practices did not significantly differ between physician groups. Falls risk, bleeding risk and poor patient adherence were all highly cited barriers to prescribing warfarin. Fewer geriatricians indicated that history of patient falls would be a reason for not treating with warfarin (G: 47%; FD: 71%; IMS: 72%), and significantly fewer changed reported practice in the presence of falls risk (*χ*^2^ (6) = 45.446, p < 0.01). Experience of a patient having a stroke whilst not on warfarin had a significant impact on vignette decisions; physicians who had had patients who experienced a stroke were more likely to prescribe warfarin (*χ*^2^ (3) =10.7, p = 0.013).

**Conclusions:**

Barriers to treatment of atrial fibrillation with warfarin affect physician specialties to different extents. Prior experience of a patient suffering a stroke when not prescribed warfarin is positively associated with intention to prescribe warfarin, even in the presence of falls risk.

## Introduction

Atrial fibrillation (AF) is a common cardiac arrhythmia, affecting 5% of the population over the age of 65 [1,2] and around 1% in individuals >20 years of age [3]. AF is a leading cause of ischemic stroke, [4] increasing the risk of stroke six-fold [5] and is estimated to account for approximately 15% of all strokes [6,7]. Consequently, management of AF involves preventing AF-related complications such as stroke.

Management of AF often relies on the administration of antithrombotic therapy. Until very recently, such therapy has chiefly taken the form of antiplatelet drugs, such as aspirin, or anticoagulants including vitamin k agonists such as warfarin. The prescription of warfarin has been shown to significantly reduce the risk of ischemic stroke [8,9]. However, the use of warfarin requires close monitoring [10] which has been and will remain an impediment to warfarin use [11,12]. Furthermore, warfarin is itself associated with a small risk of major bleeding [13]. Studies have suggested that warfarin continues to be taken by only 30–60% of appropriate patients [5,14]. A recent systematic review indicated no clear pattern of improved prescribing practice in 29 studies over a 10 year period [14]. While new oral anticoagulants (OACs), such as dabigatran, rivaroxaban, and apixaban have been developed, [4,15-18] and other agents, such as edoxaban, [19] are under review, warfarin is likely to remain an important mode of prevention of strokes in patients with AF, at least in the short- to medium-term. In part this will likely be due to the cost of the new drugs, and uncertainties around their risks in certain sub-populations such as those with poor renal function or intolerance of these new anticoagulants [18]. At the same time, attempts to improve prescribing practice through the use of genotyping prior to prescription have produced mixed results, with limited clinical utility [20]. Given these current uncertainties, improving prescribing practices for warfarin will remain an important element of clinical practice.

To date, a number of barriers to warfarin prescription have been identified. In particular, attention has been drawn to the impact of the perceived risk of the patient falling, with a concomitant risk of bleeds [2,21-23]. While some authors have suggested that the perceived risk associated with falls may be overestimated, and that it would require a substantial number of falls for the risks of falls to outweigh the benefits of warfarin prescription, [2,21] others have noted that trials of warfarin for the prevention of stroke in patients with atrial fibrillation have been highly selective in their recruitment, often excluding patients at high risk of falls or at high risk of stroke [24]. As such, it may be that physicians are forced to extrapolate from trial data that does not match the patient in front of them or rely on personal experience when making decisions in practice. While the risk of falls has been implicated in decisions not to prescribe warfarin, there has, to date, been little research attempting to assess the impact that the perceived risk of patient falls has on the decision-making of physicians when considering the prescription of warfarin for patients with atrial fibrillation.

Other work suggests prior experience with serious adverse events may also affect prescribing practice [22,25]. This has been argued to stem from the “availability heuristic”, [26] a general psychological mechanism underlying many human judgements where the judgment of a particular outcome probability can be based on the ease with which one can recall instances of similar outcomes [27]. Because vivid events are more easily recalled than mundane ones, memory of a single catastrophic bleed event while on warfarin might lead to a generalized overestimation of the probability of bleeding for subsequent patients, and consequently reduced warfarin use. Indeed, a recent meta-analysis concluded that there is a need to consider provider characteristics such as previous adverse events in patients on warfarin, and the extent to which these effects influence prescribing decisions across physician groups [28].

The aim of this study was to explore these factors that may have a disproportionate effect on warfarin treatment decisions, and to examine whether their effects vary by physician discipline.

## Methods

### Survey administration

This survey formed part of a larger project that sought to identify factors that influence physicians' decisions around the use of anti-coagulant treatment for atrial fibrillation, and focused on the use of antithrombotic treatment of AF. Physician contact details were obtained from the 2005 Canadian Medical Association Directory. The survey was administered to three samples of Canadian physicians: Family Doctors, Internal Medicine Specialists, and Geriatricians. Internal Medicine Specialists were selected as a broad physician grouping that included sub-specialties of interest, such as cardiology, but which could not be selected individually with sufficient confidence, due to variation in the way physicians were categorized. Physicians were included if they were listed as having an “active” practice status, and a specialty code of family or internal medicine. In both cases, a random sample of 500 individuals was selected. In addition to these groups, all physicians whose primary specialty was listed as geriatrics were included (n = 149).

Design and administration was optimized using the Dillman Tailored Design Method, a tested and widely used approach to survey research [29,30]. In line with this approach, physicians received an initial pre-notification letter. The survey was then mailed one week later together with a covering letter that described the purpose of the study, indicated source of funding, and reported ethical approval from the Ottawa Hospitals Research Ethics Board. Return of the survey was taken as tacit consent. Three reminders and two replacement surveys were mailed to non-responders at two-week intervals. Correspondence was addressed to the individual physicians, and signed by the principal investigator (JCB). In addition, a financial incentive ($20) was provided for completion of the survey. Surveys were completed between June and August 2007.

### Survey instrument

The survey was developed and based on a narrative review of the literature in order to gather evidence regarding physicians’ self-reported practice, reasoning around warfarin use in practice and attitudes towards previously identified barriers to the prescription of warfarin. Physicians were asked about their engagement with patients with AF, their prescribing practices when seeing a new patient with AF, the situations under which they would not prescribe warfarin, and other potential barriers to the prescription of warfarin. Potential barriers were drawn from a number of sources including the recommendations of physician organizations, CHADS_2_, [31] and the scientific literature; [7,32-34] we looked both for factors that were known to be associated outcomes related to treatment with warfarin, or those that were unrelated to outcomes, but which may be thought to be related by some physicians.

Initial instructions indicated that unless otherwise specified, all questions in the survey dealt with patients whose primary reason for seeking medical attention is AF and where AF was defined as established paroxysmal or chronic AF, involving chronic or recurrent episodes over a period of more than 48 hours. Respondents were asked to mark a line on a graded scale ranging from 0-100% to indicate the percentage of their new patients for which they would prescribe different anti-thrombotic therapies. To indicate the items deemed relevant to warfarin use, physicians were asked two questions. The first asked respondents “When you do NOT initiate warfarin (Coumadin) treatment for patients who have chronic atrial fibrillation, what reasons account for this decision?” Six options: patient judged to be of low risk of thromboembolic event (e.g., stroke); bleeding risk (e.g., liver disease); fall risk (e.g., alcohol abuse or frail older); advanced age; expected poor patient compliance in monitoring INR; and monitoring/ management logistical issues, were provided together with an open ended ‘other’ option.

To explore the role of perceived risk of bleeding on stated prescribing, three vignettes were created (Table 1). Vignettes reflected cases of low stroke and low falls risk, high stroke and low falls risk, and high stroke and high falls risk, with risk of stroke determined according to the CHADS_2_ scale [31]. To examine the potential effect of the availability heuristic on decisions of whether to prescribe warfarin, respondents were also asked about their experience of adverse events due to the prescription or non-prescription of warfarin. The questionnaire is provided in Additional file 1.

**Table 1:** Details of the case vignettes

**Vignette**	**Description:**
1	**Low Stroke risk, Low risk of falls**
	**History:** The patient is a 53 year old male, a teacher who participates in regular physical activity. He has a 5-year history of chronic atrial fibrillation which is asymptomatic. For rate control, he takes metoprolol 25 mg bid. He is not taking any antithrombotic therapy. The rest of his medical history is unremarkable, including no history of diabetes, hypertension, or other cardiovascular risk factors. He is taking no other regular medications.
	**Physical exam:** He appears fit. Blood pressure is 130/65; pulse is 78 and irregularly irregular. Cardiac exam is normal except for the irregular rhythm; and the rest of the physical exam is also normal. All laboratory work including complete blood count, electrolytes, urea, creatinine and TSH are normal. ECG confirms atrial fibrillation at 80 beats per minute, but is otherwise unremarkable. Echocardiogram (performed the next day) also shows atrial fibrillation, but is otherwise unremarkable (normal chamber sizes, normal systolic function, and no valvular abnormalities).
2	**High Stroke Risk, Low risk of falls**
	**History:** The patient is a 74 year old woman. She lives at home with her husband; she is cognitively intact (as is her husband) and is fully independent and active for her age. Past medical history includes hypertension and an ischemic stroke 4 years ago, with no residual deficits. Current medications include hydrochlorothiazide 25 mg daily and atenolol 50 mg daily. She recalled taking aspirin in the past, but stopped on her own years ago. She prefers to walk with a cane, but has never fallen.
	**Physical exam:** The patient looks well for her age. Blood pressure is 138/75, pulse is 83 but irregularly irregular. Cardiac exam is normal except for the irregular rhythm. Neurological exam is normal,
	including cranial nerves, visual fields and visual acuity. The rest of the physical exam is also normal. All laboratory work including complete blood count, electrolytes, urea, creatinine and TSH are normal. ECG confirms atrial fibrillation at 80 beats per minute, but is otherwise unremarkable. Echocardiogram (performed the next day) also shows atrial fibrillation, but is otherwise unremarkable (normal chamber sizes, normal systolic function, and no valvular abnormalities).
3	**High Stroke risk, high risk of falls**
	**History:** The patient is a 72 year old woman who lives with her husband in her own home. She is cognitively intact, as is her husband. Past history includes hypertension, and a previous ischemic stroke 3 years ago, which left her with a mild facial droop but no other neurologic deficits. She also has Parkinson’s Disease which is well controlled but has resulted in her falling 3 times in the last year. She walks with a walker. Her current medications include hydrochlorothiazide 25 mg daily, atenolol 50 mg daily, Sinemet 100/25 mg tablets three times a day. She recalled taking aspirin in the past, but stopped on her own years ago.
	**Physical Exam:** The patient has a mildly shuffling gait, but appears well for her age. Blood pressure is 139/73, pulse is 85 but irregularly irregular. Cardiac exam is normal except for the irregular rhythm. Neurological examination reveals mild left-sided lower facial weakness consistent with her past stroke and cogwheel rigidity of the extremities in keeping with her Parkinson’s. The rest of the physical exam is normal. All laboratory work including complete blood count, electrolytes, urea, creatinine and TSH are normal. ECG confirms atrial fibrillation at 86 beats per minute, but is otherwise unremarkable. Echocardiogram (performed the next day) also shows atrial fibrillation, but is otherwise unremarkable (normal chamber sizes, normal systolic function, and no valvular abnormalities).

### Data analysis

Data was entered into and analyzed using IBM SPSS Statistics v19 (SPSS Inc., 2010). A 10% data entry check was conducted by two researchers and indicated a 97.6% concordance, with discrepancies resolved through discussion and reassessment of the returned survey. Descriptive statistics were used to summarize the data from the total sample and the assessment of differences between physician groups. Comparisons between groups were made using *χ*^2^ and Kruskal-Wallis tests as appropriate; with column proportions compared using a *z* test. A level of *p* < 0.05 was accepted as significant.

## Results

Of the 1149 participants identified 117 were excluded based on the survey being ‘returned to sender’, the physician no longer being at that address, or because they did not see patients with Atrial Fibrillation. Consequently, 1032 questionnaires were included in the study (Table 2). The majority of physicians (88%) were under the age of 50 with almost two thirds (66%) of respondents being male. Assessment of non-responders was limited to the information available in the CMA Directory (i.e. sex, location and specialty). Responders did not differ significantly to non-responders on any of these variables.

**Table 2:** Demographic characteristics of the sample (n = 1032)

**Item**		**Number (%)**	
		**Responder (n = 335)**	**Non-responder (n = 697)**
Province	ON	162 (48.4)	360 (51.6)
	Other	173 (51.6)	337 (48.4)
Physician Group	Family Doctors	154 (46)	309 (44.3)
	Geriatricians	52 (15.5)	84 (12.1)
	Internal Medicine	129 (38.5)	304 (43.6)
Gender	Male	221 (65.9)	456 (65.4)
	Female	112 (33.4)	233 (33.4)
	Missing	2 (0.6)	8 (1.1)
Age group	30-39	105 (31.3)	
	40-49	110 (32.8)	
	50-59	80 (23.9)	
	60-69	30 (9)	
	70+	5 (1.5)	
	Missing	5 (1.5)	
Certified by College of Family Physicians	Yes	174 (51.9)	
	No	118 (35.2)	
	Missing	43 (12.8)	
Fellow of the Royal College of Physicians and Surgeons	Yes	184 (54.9)	
	No	117 (34.9)	
	Missing	34 (10.1)	
During the last month, how many different patients with atrial fibrillation did you manage?	0	14 (4.2)	
	1-5	130 (38.8)	
	6-10	85 (25.4)	
	10+	101 (30.1)	
	Missing	5 (1.5)	

A total of 335 surveys were returned in which physicians reported seeing at least one patient with atrial fibrillation per year (response rate 32.5%). Of these, 154 (46%) were family doctors, 129 (38.5%) internal medicine specialists, and 52 (15.5%) were geriatricians.

### Standard AF treatment practice

Table 3 presents physician responses regarding the percentage of patients with atrial fibrillation to whom they report administering anti-thrombotic therapies. Warfarin was the most common treatment with a mean of 73% of physicians indicating this would be the drug they administered to new patients. There was no statistically significant variation between physician types in terms of the reported rates of warfarin use (p =0.28), no antithrombotic therapy (p = 0.21), aspirin (p = 0.24), Plavix (Clopidogrel) (p = 0.11), or other treatments (p = 0.46).

**Table 3:** Estimated mean percentages of new patients treated with defined anti-thrombotic therapies

	**When you see new patients with atrial fibrillation, for what percentage do you administer the following anti-thrombotic therapies?**				
	**All (n = 331)**	**Geriatricians (n = 51)**	**Family doctors (n = 151)**	**Internal Medicine Specialists (n = 129)**	**P-value**^**§**^
Warfarin	72.6%	76.4%	71.9%	71.9%	0.28
no antithrombotic therapy	5.4%	4.9%	5.3%	5.8%	0.21
Aspirin	20.7%	19.8%	20.4%	21.5%	0.24
Plavix (Clopidogrel)	4.2%	3.8%	5.2%	3.2%	0.11
Other	2.9%	1.6%	2.7%	3.6%	0.46

^§^Kruskal-Wallis; P < 0.05.

### Reasons for non-prescription of warfarin

Table 4 shows the reasons reported for not prescribing warfarin, stratified by physician group. There were statistically significant differences between physician groups for all factors with the exception of the ‘other’ category. Generally, geriatricians differed in their responses to the other two disciplines. A significantly lower proportion of geriatricians indicated that risk of falls would be a reason not to prescribe warfarin, (geriatricians (G):47%; family doctors (FD): 71%; and internal medicine specialists (IMS): 72%). This was also the case for advanced age (G: 7.8%; FD: 31.1%; IMS: 25.8%). Geriatricians also indicated high levels of concern regarding expected poor patient adherence (G: 74.5%; FD: 49%; IMS: 66.4%), although this did not significantly differ from internal medicine specialists.

**Table 4:** Physician reasons for not prescribing Warfarin (n = 330)

	**When you do NOT initiate Warfarin (Coumadin) treatment for patients who have chronic atrial fibrillation, what reasons account for this decision?**				
	**All**	**Geriatricians (n = 51)**	**Family doctors (n = 151)**	**Internal Medicine Specialists (n = 128)**	**P-value**^**§**^
Patient judged low risk	55.5%	25.5%_a_	51.7%_b_	71.9%_c_	<0.01
Bleeding risk	67.3%	52.9%_a_	63.6%_a_	77.3%_b_	<0.01
Fall risk	68.2%	47.1%_a_	71.5%_b_	72.7%_b_	<0.01
Advanced age	25.5%	7.8%_a_	31.1%_b_	25.8%_b_	<0.01
Expected poor patient compliance	59.7%	74.5%_a_	49%_b_	66.4%_a_	<0.01
Monitoring/management logistical issues	26.4%	52.9%_a_	14.6%_b_	29.7%_c_	<0.01
Other	14.5%	19.6%_a_	13.2%_a_	14.1%_a_	0.53

^**§**^Kruskal-Wallis; P < 0.05. Subscripts denote a subset of physician group categories whose column proportions are similar at the P = 0.05 level. For example, columns both containing the subscript ‘a’ are similar, but can be differentiated from columns containing b. Columns containing ‘a,b’ cannot be differentiated from columns containing ‘a’ or ‘b’.

Falls risk, bleeding risk and poor patient adherence were all highly cited reasons for not treating with warfarin. A significantly higher proportion of internal medicine specialists indicated that bleeding risk would be a barrier to the prescription of warfarin. Attitudes to monitoring and management issues differed across all three physician types with 53% of geriatricians indicating monitoring or management issues as reasons for non-prescription compared with only 15% of family doctors and 30% of internal medicine specialists.

Table 5 describes the reported experience of adverse events among the three physician groups. Overall, bleeds had been more commonly experienced (60.3%) than strokes (31.4%). Internal medicine specialists were the most likely to report having seen both kinds of adverse events (Bleed 72.7%; Stroke 41.9%), and family physicians the least likely (Bleed 49.7%; Stroke 21.5%).

**Table 5:** Physician experience of adverse events of serious bleed or stroke in patients

**Experience**					
	**All**	**Geriatricians**	**Family doctors**	**Internal Medicine Specialists**	**P-value**^**§**^
Ever had a patient suffer a serious bleed (% Yes) (n = 330)	60.3%	60.8%_a,b_	49.7%_a_	72.7%_b_	<0.01
Have you ever had a patient suffer a serious stroke (% Yes) (n = 322)	31.4%	34.7%_a,b_	21.5%_a_	41.9%_b_	<0.01

^§^*χ*^2^. Each subscript letter denotes a subset of physician group categories whose column proportions are similar at the P = 0.05 level. For example, columns both containing the subscript ‘a’ are similar, but can be differentiated from columns containing b. Columns containing ‘a,b’ cannot be differentiated from columns containing ‘a’ or ‘b’.

### Prescribing responses to vignettes

**Figure 1:**
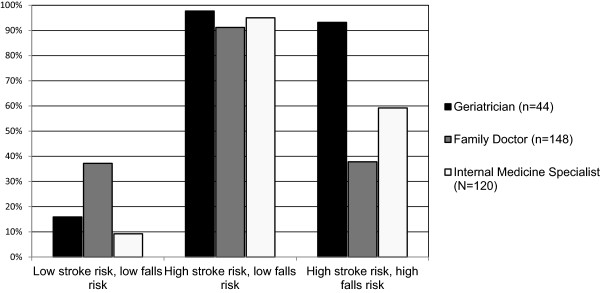
**Stated rates of treatment with conventional warfarin for each scenario (n = 312).** Percentage of physicians reporting that they would prescribe conventional warfarin (INR 2.0-3.0) for each of the scenarios presented in Table 1.

Warfarin prescription decisions were made by respondents for three hypothetical patients 1) low stroke risk, low falls risk; 2) high stroke risk, low falls risk; 3) high stroke risk, high falls risk; data are shown in Figure 1. The third vignette described a patient with a similar high stroke risk as in vignette 2, but with the addition of a risk of falls (3 times in the last year). Such occasional falls are not a contraindication of warfarin prescription.

Warfarin use was lowest for the first vignette, although physician groups varied significantly (G: 15.9%; FD: 37.2%; IMS: 9.2%). For a patient with a high stroke risk and low falls risk, warfarin use was universally high, and did not differ between groups (G: 97.7%; FD: 91.2%; IMS: 95%).

Physician groups differed dramatically on whether the addition of a falls risk would change their decision to prescribe warfarin. Almost all (93.2%) of geriatricians reported that they would prescribe warfarin for such a patient; this did not differ significantly from the proportion that would give warfarin to a similar patient without the falls risk (97.7%). However, only 59.2% of IMSs and 37.8% of FDs would prescribe warfarin, compared to 95% and 91.2% respectively for patients without the risk of falls; this difference between physician groups was significant (Fisher’s exact test p < 0.01, Pearson *χ*^2^(6) = 45.446, p < 0.01). Stated another way, while only 4.5% of geriatricians reported a different warfarin prescription decision between vignettes 2 and 3, the number was 36.7% for Internal Medicine Specialists and 54.1% for Family Physicians.

Finally, we combined the data from the three physician groups to examine whether prior experience with a patient having had a stroke or bleed would be related to reduced likelihood of warfarin given an occasional risk of falls i.e. change in practice between vignettes two and three. Experience with having seen a serious bleed was not associated with that likelihood (*χ*^2^ (3) =3.039, p = 0.386, Fisher’s exact test p = 0.319, n = 308). However, experience with having seen a stroke in a patient not prescribed warfarin was a significant influence; those who had a patient experience a stroke while not receiving warfarin were more likely to persist with, or introduce, the prescription of warfarin in the presence of risk of falls (*χ*^2^(3) = 10.7, p = 0.013, Fisher’s exact test p = 0.013, n = 301).

## Discussion

Consistent with previous research, [11,28,35-40] the present study observed variation in reported prescribing practices for atrial fibrillation and the identification of variables such as perceived bleeding risk, fall risk, and expected poor patient adherence as commonly cited factors that would decrease physicians’ likelihood of prescribing warfarin. While previous studies have indicated the role of factors such as perceived risk of falls as a barrier to warfarin prescription, [2,21-23] we further this research by analyzing the impact on prescribing decisions across different physician populations.

Our results demonstrated a high reported used of warfarin, with all physician groups stating that they prescribe warfarin to 70-75% of their patients with atrial fibrillation. This suggests that there is no one group of physicians that is wildly different in their planned use of the drug, either because of a lack of knowledge about outcomes, or relevant practice differences. These rates must, however, be cautiously interpreted given the self-reported nature and are above the typical estimates of current warfarin use, which have been reported to be as low as 19%, but as high as 78% [11,28,32]. However, a strength of the present study is the comparisons made across different physician specialties and the reported differences in reasons for not prescribing warfarin. Specifically we identified a differential effect of risk of falls on prescribing decisions.

Several potential limitations of this study warrant consideration. Our response rate was only 32.5%, although this is similar to previous surveys with physicians regarding treatment practices [37,38,41]. We attribute this in part to the length of the survey, which was six pages in length and required some effort to complete. While there were no clear differences on available variables between responders and non-responders, and response rate was similar across physician groups, our limited knowledge about non-responders leaves open the possibility of a response bias. A second limitation is that the data were collected in 2007, and since then a number of new alternatives to warfarin have become available. However, a number of questions remain over the use of new therapies due to (1) the significantly increased costs over warfarin and (2) the concern regarding the treatment of side effects – such as bleeding. Until these issues have been addressed warfarin is likely to remain an important treatment for atrial fibrillation.

Our results indicate that physician groups differ in terms of the barriers reported to affect warfarin-prescribing decisions. While previous research identified geriatricians as being more likely to indicate warfarin prescription than other physicians types, [42] it only compared responses to two scenarios that varied in several aspects. Our results provide increased clarity on this issue and suggest that geriatricians seem to consider bleeding risk, risk of falls, and advanced age as less influential on prescribing decisions than the other physician groups. Similarly, family doctors were less likely than geriatricians to indicate they would be affected by logistical issues such as poor patient adherence and monitoring issues. One suggestion may be that family doctors may be more likely to refer these patients to other specialties and so may be less likely to experience adherence issues. Whether these group differences can be best explained by experience, training, or as a reflection of the capabilities of their respective patient populations requires further investigation. The subsequent development of tools such as HAS-BLED, [43] HEMORR2HAGES, [44] and ATRIA [45] for the assessment of bleeding risk – and which explicitly incorporate advanced age – may assist standardizing risk assessments for bleeding as part of the clinical assessments as to the net clinical benefit of oral anti-coagulants, [46] although there remains an ongoing need for assessment of prospective clinical utility.

Prior experience of patients suffering adverse events also appeared to affect prescribing practice. Aggregated physician data indicated a positive association between experience of a patient suffering a stroke when not prescribed warfarin and an indication that they would continue or instigate prescribing of warfarin in the presence of falls risk. We did not, however, find an association between experience of adverse bleeding and warfarin prescription in the presence of falls risk. This finding is consistent with several other survey-based studies [22,41]. Using a cross-sectional survey of physicians within the American Medical Association, Gross et al., [41] reported more physicians to experience regret over acts of omission (not prescribing warfarin and then stroke) than commission (prescribing warfarin then bleed) and an associated increase in stated warfarin prescription when presented with a range of case vignettes [41]. This accords with Australian data from family physicians [22] in which one fifth felt responsible for acts of commission leading to hemorrhage, whilst just under a third reported they would feel responsible for an act of omission that lead to an ischemic stroke. However, in a study of actual prescribing and past patient bleeding/stroke, an association between physician experience of serious bleeding and reduced warfarin prescription was found [25]. This may point to a disagreement between self-reported and actual behavior which indicates an important area requiring further study.

Overall, and consistent with previous results, [22] we saw a general drop in the proposed use of conventional warfarin when the risk of recurrent falls was introduced within the vignettes. However, an intriguing result was the clear differences in how the different physician groups incorporate falls risk into their decisions. Inclusion of falls risk into an otherwise comparable, high stroke risk patient resulted in avoidance of warfarin in 37% of Internal Medicine Specialists, 54% of Family Physicians, and only 5% of Geriatricians. While older patients have not been included in clinical trials, and so one might expect geriatricians to be more conservative in their use of warfarin, geriatricians may have greater clinical experience with how warfarin might affect older patients through increased exposure to atrial fibrillation. They may, therefore, be basing their decisions on this clinical experience as opposed to trial data. Whether such differences in the stated use of warfarin in the presence of falls can be attributable to reasonable differences in practice, or whether it points to a misunderstanding of the risk associated with falls in one or more physician group is an important area for further research, particularly given the lack of trial data relating to older patients, those at increased risk of falls, and at higher risk of stroke.

## Conclusion

Our study provides a number of insights into self-reported physician prescribing practice and the barriers to prescribing warfarin. Our results replicate previous studies and identify a number of potential barriers to warfarin prescription, but have identified differing impact across physician populations. In particular, we note the variation regarding adverse events on stated prescribing practice; prior experience of a patient suffering a stroke when not prescribed warfarin was positively associated with an intention to prescribe warfarin, even in the presence of falls risk. The indication that physicians of differing specialty don’t report significantly different rates of practice may indicate that general educational interventions will be ineffective. Rather, specific patient- or practice-level feedback may be appropriate. Harnessing the potential of electronic medical records and techniques such as audit and feedback, [47,48] may provide ‘personalized’ feedback on clinical practice that will be both more salient and informative with respect to monitoring prescribing practice for warfarin [49]. Our results provide insight into where variation in physician decision making occurs and may point to relevant elements to be included in such feedback. Despite this suggestion, there remains a need for further research to explore how physicians weight specific indicators under different scenarios and to investigate how new agents will modify decisions given the notable impact of monitoring on physician practice.

## Competing interests

There are no conflicts of interests to declare.

## Authors’ contributions

SGN conducted the primary analyses, and led the writing of the paper; JCB designed the study, wrote the study proposal, protocol, and methodology. RGA, KC, and RP provided additional analyses and contributed to the writing of the manuscript. RMP conceived the general research questions. RMP, KGS, and JG provided specific clinical and/or methodological expertise. All authors contributed to read and approved the final manuscript.

## Supplementary Material

Additional file 1 Survey of Canadian Physicians – Use of anti-thrombotic therapy for Atrial Fibrillation. Click here for file
