# Supplementary material for: Impact of stated barriers on proposed warfarin prescription for atrial fibrillation: a survey of Canadian physicians
Source: Thromb J. 2014 Jun 23;12:13. doi: 10.1186/1477-9560-12-13 (PMC4144316; doi:10.1186/1477-9560-12-13)
Supplement: Additional file 1 — Survey of Canadian Physicians – Use of anti-thrombotic therapy for Atrial Fibrillation. [file 1477-9560-12-13-S1.pdf]

Fax to (613) 761-5402 ID#I
«Doc_Num»

OTTAWA HEALTH RESEARCH INSTITUTE
INSTITUT DE RECHERCHE EN SANTÉ D'OTTAWA

Survey of Canadian Physicians – Use of anti-thrombotic therapy for Atrial Fibrillation

On the following pages are a number of questions asking about the conditions under which you would prescribe anticoagulation therapy.

Please note that unless otherwise specified, all questions deal with patients whose primary reason for seeking medical attention is atrial fibrillation.

All responses will be kept in strict confidence and for research purposes only.

Please return to Jamie Brehaut in the envelope provided or fax to (613) 761-5402.

CIHR IRSC

Fax to (613) 761-5402 ID#I
«Doc_Num»

OTTAWA HEALTH RESEARCH INSTITUTE
INSTITUT DE RECHERCHE EN SANTÉ D'OTTAWA

Section 1: Current Practice

Throughout this survey, 'atrial fibrillation' refers to established paroxysmal or chronic atrial fibrillation, involving chronic or recurrent episodes over a period of more than 48 hours.

Do you manage at least one patient with atrial fibrillation per year in your current practice?
☐ Yes
☐ No *(If no, please proceed to Section 4 on final page)*

During the last month, how many different patients with atrial fibrillation did you manage?
☐ 0
☐ 1-5
☐ 6-10
☐ 10+

When you see new patients with atrial fibrillation, for what percentage do you administer the following anti-thrombotic therapies? *(mark a line at the appropriate percentage; total should approximately equal 100%).*

	0%	20	40	60	80	100%
No anti-thrombotic therapy	|---------|---------|---------|---------|---------|---------|---------|---------|---------|---------|					
Aspirin	|---------|---------|---------|---------|---------|---------|---------|---------|---------|---------|					
Plavix (Clopidogrel), with or without Aspirin	|---------|---------|---------|---------|---------|---------|---------|---------|---------|---------|					
Warfarin (Coumadin)	|---------|---------|---------|---------|---------|---------|---------|---------|---------|---------|					
Other; ________________________________	|---------|---------|---------|---------|---------|---------|---------|---------|---------|---------|					

When you do NOT initiate Warfarin (Coumadin) treatment for patients who have chronic atrial fibrillation, what reasons account for this decision? *(you may check off more than 1)*
☐ patient judged to be of low risk of thromboembolic event (e.g., stroke)
☐ bleeding risk (e.g., liver disease)
☐ fall risk (e.g., alcohol abuse or frail older)
☐ advanced age
☐ expected poor patient compliance in monitoring INR
☐ monitoring/ management logistical issues
☐ other; please specify____

Have you ever had a patient who suffered a serious bleeding complication while on Warfarin therapy you prescribed?
☐Yes; please specify ☐Fatal bleed or intracranial hemorrhage
☐Other bleed requiring hospitalization
☐Other; please specify____

Over the past 10 years, approximately how often have such serious bleeds occurred? _______ *times*

How long ago was the most recent event of this type? ☐ Years ☐ Months ☐ Weeks ☐ Days

☐ No

Have you ever had a patient who suffered a serious stroke after you had chosen NOT to prescribe Warfarin therapy?
☐Yes; please specify ☐Fatal or permanently disabling stroke
☐Non-disabling stroke or transient ischemic attack; please specify______________________________

Over the past 10 years, approximately how often has this occurred? _______ *times*

How long ago was the most recent event of this type? ☐ Years ☐ Months ☐ Weeks ☐ Days

☐ No

Section 2: Patient Vignettes

On the following pages are three patient vignettes. Please read through these vignettes and answer the questions as best you can, based on your training, experience, and knowledge. Please approach these vignettes as if they represent patients you are seeing <u>for the first time</u> in your practice, presenting for a general assessment. Even if you would refer the following patients to a specialist, please complete the questions as though they are patients you would treat in your practice. <u>Please assume that all patients below are non-smokers who drink only occasionally, and for whom no other diseases exist.</u>

Case # 1 of 3

<u>History</u>
The patient is a 53 year old male, a teacher who participates in regular physical activity. He has a 5-year history of chronic atrial fibrillation which is asymptomatic. For rate control, he takes metoprolol 25 mg bid. He is not taking any antithrombotic therapy. The rest of his medical history is unremarkable, including no history of diabetes, hypertension, or other cardiovascular risk factors. He is taking no other regular medications.

<u>Physical exam</u>
He appears fit. Blood pressure is 130/65; pulse is 78 and irregularly irregular. Cardiac exam is normal except for the irregular rhythm; and the rest of the physical exam is also normal. All laboratory work including complete blood count, electrolytes, urea, creatinine and TSH are normal. ECG confirms atrial fibrillation at 80 beats per minute, but is otherwise unremarkable. Echocardiogram (performed the next day) also shows atrial fibrillation, but is otherwise unremarkable (normal chamber sizes, normal systolic function, and no valvular abnormalities).

A. Please indicate your preferred choices of anti-thrombotic therapy for this patient.

☐No anti-thrombotic therapy ☐Aspirin 325 mg daily ☐Clopidogrel (Plavix) ☐Low-intensity warfarin (INR <2) ☐Conventional warfarin (INR 2.0-3.0) ☐Other specify__________

B. <u>Regardless of how you would treat the above patient</u>, please estimate how often 100 patients of this kind would experience stroke* or major hemorrhage* given the listed treatment. *If you are unfamiliar with any treatment leave that row blank.*

Assume 100 patients like the one above.

1a) If all 100 patients received **no antithrombotic therapy,** how many would have a stroke* over the next year? __________
1b) How many of the 100 would have some form of major hemorrhage*? __________

2a) If all 100 received **Warfarin therapy,** how many would have a stroke over the next year ?__________
2b) How many of the 100 would have some form of major hemorrhage? __________

3a) If all 100 received **Aspirin,** how many would have a stroke over the next year ?__________
3b) How many of the 100 would have some form of major hemorrhage? __________

4a) If all 100 received **Clopidiogrel (Plavix),** how many would have a stroke over the next year ?__________
4b) How many of the 100 would have some form of major hemorrhage? __________

** Disabling ischemic strokes are those with functional impairment affecting basic activities of daily living.*
** Major hemorrhage refers to any bleed causing death or requiring hospitalization.*

Case # 2 of 3 **Please complete all three vignettes, it is very important!**

History

The patient is a 74 year old woman. She lives at home with her husband; she is cognitively intact (as is her husband) and is fully independent and active for her age. Past medical history includes hypertension and an ischemic stroke 4 years ago, with no residual deficits. Current medications include hydrochlorothiazide 25 mg daily and atenolol 50 mg daily. She recalled taking aspirin in the past, but stopped on her own years ago. She prefers to walk with a cane, but has never fallen.

Physical exam

The patient looks well for her age. Blood pressure is 138/75, pulse is 83 but irregularly irregular. Cardiac exam is normal except for the irregular rhythm. Neurological exam is normal, including cranial nerves, visual fields and visual acuity. The rest of the physical exam is also normal. All laboratory work including complete blood count, electrolytes, urea, creatinine and TSH are normal. ECG confirms atrial fibrillation at 80 beats per minute, but is otherwise unremarkable. Echocardiogram (performed the next day) also shows atrial fibrillation, but is otherwise unremarkable (normal chamber sizes, normal systolic function, and no valvular abnormalities).

A. Please indicate how you would deal with this patient with regard to anti-thrombotic therapy.

☐No anti-thrombotic therapy ☐Aspirin 325 mg daily ☐Clopidogrel (Plavix) ☐ Low-intensity warfarin (INR <2) ☐ Conventional warfarin (INR 2.0-3.0) ☐Other specify__________

B. <u>Regardless of how you would treat the above patient</u>, please estimate how often 100 patients of this kind would experience stroke or major hemorrhage given the listed treatment. *If you are unfamiliar with any treatment leave that row blank.*

Assume 100 patients like the one above.

1a) If all 100 patients received **no antithrombotic therapy,** how many would have a stroke over the next year? __________
 1b) How many of the 100 would have some form of major hemorrhage? __________

2a) If all 100 received **Warfarin therapy,** how many would have a stroke over the next year ?__________
 2b) How many of the 100 would have some form of major hemorrhage? __________

3a) If all 100 received **Aspirin,** how many would have a stroke over the next year ?__________
 3b) How many of the 100 would have some form of major hemorrhage? __________

4a) If all 100 received **Clopidiogrel (Plavix),** how many would have a stroke over the next year ?__________
 4b) How many of the 100 would have some form of major hemorrhage? __________

Case # 3 of 3

History

The patient is a 72 year old woman who lives with her husband in her own home. She is cognitively intact, as is her husband. Past history includes hypertension, and a previous ischemic stroke 3 years ago, which left her with a mild facial droop but no other neurologic deficits. She also has Parkinson's Disease which is well controlled but has resulted in her falling 3 times in the last year. She walks with a walker. Her current medications include hydrochlorothiazide 25 mg daily, atenolol 50 mg daily, Sinemet 100/25 mg tablets three times a day. She recalled taking aspirin in the past, but stopped on her own years ago.

Physical Exam

The patient has a mildly shuffling gait, but appears well for her age. Blood pressure is 139/73, pulse is 85 but irregularly irregular. Cardiac exam is normal except for the irregular rhythm. Neurological examination reveals mild left-sided lower facial weakness consistent with her past stroke and cogwheel rigidity of the extremities in keeping with her Parkinson's. The rest of the physical exam is normal. All laboratory work including complete blood count, electrolytes, urea, creatinine and TSH are normal. ECG confirms atrial fibrillation at 86 beats per minute, but is otherwise unremarkable. Echocardiogram (performed the next day) also shows atrial fibrillation, but is otherwise unremarkable (normal chamber sizes, normal systolic function, and no valvular abnormalities).

A. Please indicate how you would deal with this patient with regard to anti-thrombotic therapy.

☐No anti-thrombotic therapy ☐Aspirin 325 mg daily ☐Clopidogrel (Plavix) ☐ Low-intensity warfarin (INR <2) ☐ Conventional warfarin (INR 2.0-3.0) ☐Other specify__________

B. Regardless of how you would treat the above patient, please estimate how often 100 patients of this kind would experience stroke or major hemorrhage given the listed treatment. *If you are unfamiliar with any treatment leave that row blank.*

Assume 100 patients like the one above.

1a) If all 100 patients received **no antithrombotic therapy,** how many would have a stroke over the next year? __________
1b) How many of the 100 would have some form of major hemorrhage? __________

2a) If all 100 received **Warfarin therapy,** how many would have a stroke over the next year ?__________
2b) How many of the 100 would have some form of major hemorrhage? __________

3a) If all 100 received **Aspirin,** how many would have a stroke over the next year ?__________
3b) How many of the 100 would have some form of major hemorrhage? __________

4a) If all 100 received **Clopidiogrel (Plavix),** how many would have a stroke over the next year ?__________
4b) How many of the 100 would have some form of major hemorrhage? __________

Fax to (613) 761-5402 ID#I
«Doc_Num»

Section 3: Other Factors

In general which of the following factors individually would decrease the likelihood you would prescribe Warfarin?

Patient Medical Characteristics
- ☐ Serious or recurrent bleeding in past
- ☐ Cirrhosis or alcohol abuse
- ☐ Concurrent NSAID use
- ☐ Medications or herbal products that interact with Warfarin
- ☐ Infrequent, brief episodes of AF
- ☐ Fall Risk (falls daily)
- ☐ Fall Risk (falls monthly)
- ☐ Fall Risk (falls yearly)
- ☐ Younger Age
- ☐ Stroke prophylaxis not primary concern in patient's overall treatment goals (e.g. very poor prognosis)
- ☐ Other ____________________

Patient Capabilities
- ☐ Difficulty contacting patient for dose changes
- ☐ Difficulty for patient to attend lab appointments
- ☐ Difficulty to comprehend or adhere to relevant instructions or restrictions
- ☐ Treatment cost in general
- ☐ Other ____________________

Patient Preference
- ☐ Stroke prophylaxis a low priority for patient
- ☐ Patient concerned about risk of bleeding
- ☐ Patient finds lifestyle changes difficult
- ☐ Patient unwilling to comply with monitoring requirements
- ☐ Other ____________________

Section 4: About You

The following questions are about your background and practice setting

Are you: ☐ Male ☐Female

Your age range:
- ☐ 20-29
- ☐ 30-39
- ☐ 40-49
- ☐ 50-59
- ☐ 60-69
- ☐ 70+

Year of graduation from medical school: ________
Country of graduation from medical school: ___________________________

What is your specialty? *(check one)*
- ☐ Family Practice
- ☐ Geriatric Medicine
- ☐ Internal Medicine
- ☐ Other _____________________

What is your practice setting?*(check one)*
- ☐ Hospital
- ☐ Community

What is your practice type?*(check one)*
- ☐ Solo Practice
- ☐ Single specialty group
- ☐ Multi- specialty group

	Yes	No
**Are you certified by The College of Family Physicians of Canada?**	☐	☐
**Are you a Fellow of The Royal College of Physicians and Surgeons of Canada?**	☐	☐

Thank you for completing our survey! Should we

ID#

☐Send the $20 to you
☐Send the $20 to charity (☐Doctors without Borders, ☐ Heart and Stroke Foundation)
☐ Provide you with a tax receipt (sent to same address; we will request ‘no further solicitation’)

Please return in the envelope provided or Fax to (613) 761-5402.
